# Executive Function, Chaos and Temperament: Specificities in Preschoolers with Externalizing Behaviors

**DOI:** 10.5334/pb.352

**Published:** 2018-08-22

**Authors:** Alexandra Volckaert, Marie-Pascale Noël

**Affiliations:** 1 Université Catholique de Louvain, Institute of Psychological Sciences, 10, Place Cardinal Mercier, 1348 Louvain-la-Neuve, BE

**Keywords:** Executive function, preschoolers, externalizing behaviors, chaos, temperament

## Abstract

Various factors may contribute to the emergence of externalizing behavior (EB) problems in the preschool period. At the child level, temperament and executive function (EF) seem to play an important role, as well as environmental variables such as household chaos. In this study, we examined the profiles of 49 EB preschoolers compared to 49 typically developing (TD) preschoolers matched on age and gender. To evaluate the behavioral aspect of EB, we asked teachers and parents to fill out questionnaires, but we also used an observational paradigm. We assessed executive functions using attention, inhibition, flexibility and working memory tests. Finally, we used questionnaires to assess household chaos and child temperament. Results showed that children rated by parents as presenting EB were also assessed so by teachers and exhibited more agitation in our observational paradigm. As expected, EB children also presented weaker performance than the TD children in all EF tasks, except those measuring attention, and showed a larger reaction-time variability. Parents of the EB group reported a more chaotic environment at home. Finally, we found that child temperament (i.e., emotionality) also plays a role in group belonging. This study shows that EB children already exhibit specific characteristics by the time they are of preschool age, not only in the behavioral sphere, but also in the cognitive and environmental areas. However, despite all the differences between the two groups, a discriminant analysis showed that EF capacities have a weak power for EB diagnosis.

## Introduction

‘Externalizing behavior problems’ (EB) refers to problematic behaviors which are directed towards others, such as physical aggression (e.g., hitting, biting, shoving others), verbal aggression (e.g., teasing, threats) or disruptive behavior (e.g., tantrums, disobedience, agitation, inattention, failure to comply with limits). In young children, these behaviors are often the reason for consultation with health professionals such as psychologists, pediatricians and child psychiatrists (Hinshaw & Anderson, 1996; Smeekens, Riksen-Walraven, & van Bakel, 2007). Parents regularly report that their child is difficult at home and that it is hard for them to manage these disruptive behaviors. However, in young children, it is not easy to determine whether these behaviors should be considered as pathological or as a part of normal child development. Indeed, EB problems lie on a continuum from normal to pathological and a certain level of EB can be considered as typical in young children (Wakschlag et al., 2007).

It seems clear nowadays that various factors contribute to the emergence of EB problems (Cohen, 2008; Smeekens et al., 2007). In 1998, Deater-Deckard, Dodge, Bates and Pettit highlighted multiple risk factors in the development of EB in unselected 5- to 10-year-old children. They measured 20 risk variables and found that 18 of these correlated with EB. These risk variables were related to four domains: *parenting and caregiving* such as harsh discipline, physical harm, poor involvement of biological father, parental conflict or exposure to violence; *peer experiences* such as peer rejection; *sociocultural risks* such as poverty, structural characteristics of family or social isolation; and *child risk factors* such as medical problems in childhood, gender or temperament.

Roskam, Meunier, Stievenart, and Noël (2013) reported a more detailed analysis of four risk factors that play a role in the onset of EB at preschool age. They highlighted two risk factors at the family level and two risk factors at the child level. At the family level, insecurity and disorganization of attachment together with negative control used by parents in education (harsh and inconsistent punishment, high coercion, etc.) appear to be related to the development of EB in young children (see Mervielde, De Clercq, De Fruyt, and Van Leeuwen (2005), M. A. Barnett, Shanahan, Deng, Haskett, and Cox (2010); Brocki and Bohlin (2006); Fearon, Bakermans-Kranenburg, Van IJzendoorn, Lapsley, and Roisman (2010) for similar observations).

At the child level, they underlined the role of the child’s temperament and of poor inhibition capacities. The link between temperament and EB is also well established in the literature (Sanson, Hemphill, & Smart, 2004; Shaw, Owens, Giovannelli, & Winslow, 2001). For instance, Meunier et al. (2011) showed a significant negative correlation between child emotional stability, conscientiousness, and agreeableness and the presence of EB in preschoolers. Schmitz et al. (1999) showed that high emotionality evaluated at 14 months of age predicted high EB later on at 20, 24 and 36 months. Rende (1993) also found that emotionality was the most predictive temperament trait for behavior problems.

Many studies have also shown the association between EB and poor executive functions (EF) in the preschool period. Indeed, both studies on typically developing (TD) preschoolers and studies comparing TD and EB preschoolers have repeatedly shown correlations between EB and EF (Livesey, Keen, Rouse, & White, 2006; Raaijmakers et al., 2008; Schoemaker, Bunte, Espy, Deković, & Matthys, 2014). Two meta-analyses have recently been published about this topic. In the first, Pauli-Pott and Becker (2011) reviewed 25 studies testing the association between EF performance and the presence of ADHD symptoms (hyperactivity, impulsivity and inattention) in preschoolers. They showed a high correlation between ADHD symptoms and attention-vigilance measures (*r* = .27), interference control (*r* = .26, e.g., Stroop test) and inhibition (*r* = .29, e.g., in a Go-NoGo task), but the correlation was weak with flexibility and working memory measures. In the second meta-analysis, Schoemaker, Mulder, Deković, and Matthys (2013) focused not only on studies in preschoolers showing symptoms of ADHD but also of those with oppositional defiant disorders. They also found that EB was related to an overall EF factor^1^ (*r* = .22) and more specifically to inhibition (*r* = .24), whereas effect size was smaller for working memory (*r* = .17) and flexibility (*r* = .13). They did not, however, investigate the link with attention capacities. Other longitudinal studies in TD populations have shown that inhibition capacities at preschool age are a significant predictor of EB one year later (Hughes & Ensor, 2008), two years later (Bohlin, Eninger, Brocki, & Thorell, 2012) or even three years later (Berlin, Bohlin, & Rydell, 2003). More recently, the causal link between poor EF and EB has been sustained by a training study showing that enhancing EF in preschoolers reduced their EB (Volckaert & Noël, 2015).

Several studies also showed that ADHD school-aged children or adults often present more variability in their reaction times in computer cognitive tasks than do control participants. This increased variability has been found both in tasks involving executive components and in those that do not (for more details see the meta-analysis by Kofler et al. (2013) or the review of Tamm et al. (2012)). According to Kofler et al. (2013), this variability actually reflects attentional lapses during the task. As far as we know, this aspect has not been studied in the preschool population.

Moreover, there is evidence that home environment may also contribute to EB. Matheny, Wachs, Ludwig, and Phillips (1995) created the *Confusion, Hubbub, and Order Scale* (CHAOS) questionnaire, which evaluates household chaos. They found that a noisy, crowded and confused environment is negatively correlated with developmental variables such as cognitive performance, academic achievement, language, motivation, temperament, and cooperative play. Moreover, such an environment may also indirectly influence the child’s development through the parents’ behavior. Indeed, in a tiring, noisy environment, the parent is more likely to be less responsive, to talk less, and to provide less scaffolding (which is so important in helping children to develop new skills). Corapci and Wachs (2002) also reported that children who live in more chaotic homes have a more negative mood, have more intense reactions and a temperament that is more difficult to manage. This is confirmed by the recent study by Farbiash, Berger, Atzaba-Poria, and Auerbach (2014) which showed that household chaos is associated with negative outcomes in young children such as aggression, emotional problems, conduct problems, and hyperactivity. Chaos also seems to be negatively correlated with self-regulation, which in turn correlates with EB. Deater-Deckard et al. (2009) showed in a longitudinal study with TD children that the CHAOS score predicts EB three years later.

Some researchers have also highlighted a link between chaos and EF. In 2009, Hughes and Ensor showed that household chaos was negatively associated with the development of EF between ages 2 and 4. Hence more and more researchers are interested in the risk factors thought to be involved in the emergence of EB in young children. Yet most of these studies have considered these risk factors separately. To our knowledge, only two studies considered a larger picture; they showed that the greater the number of risk factors, the greater the likelihood of developing EB (Deater-Deckard et al., 1998; Roskam et al., 2013).

In this study, we wanted to consider a series of risk factors or characteristics of EB taken together in preschool children. In particular, we considered three dimensions that have been shown to be related in EB in preschool children: EF capacities, reaction time variability (RTV), as well as child temperament and family chaos. Two groups of preschool children were compared, one presenting a critical rate of EB that led their parents to complain about their child and the other not. Existing studies on EB and risk factors are generally composed of unselected samples, using EB problems as a continuum (Berlin & Bohlin, 2002; Berwid et al., 2005; Raaijmakers et al., 2008). When authors include clinical samples, the two compared groups are not always exactly similar in terms of socio-economic status (SES) or parental education (Brophy, Taylor, & Hughes, 2002; Hughes, Dunn, & White, 1998), or even IQ (Mariani & Barkley, 1997). Here, the group of children with a critical level of EB (ED group) was compared with a group of typically developing children (TD group) that had comparable age, gender, SES, parental education and IQ. Moreover, in most of these studies, EB was assessed through questionnaires filled out by parents as well as, sometimes, by teachers (Owens & Shaw, 2003; Smeekens et al., 2007). In this study, we wanted to keep these observers’ assessments but we also wanted to have a direct objective measure of the child’s EB in a controlled environment. To that end, we included an observational paradigm which included a situation inducing frustration (Roskam et al., 2016). Concerning EF measures, we used a questionnaire filled out by the child’s parents and by the teacher to get a subjective measure of the child’s capacities in the ecological environment as well as a large battery of cognitive tests measuring the child’s attention, working memory, flexibility and inhibition capacities, with a stronger emphasis on the last one as it is known to be the EF which is most strongly related to EB. We also measured attentional lapses through the RTV. Hence, in this study, we compared the EB and the TD samples of children on the different dimensions that were measured: EF, RTV, temperament and chaos. We expected EB children to have poorer EF capacities than TD children, especially in inhibition tests, and to present greater RTV as well. We also expected the EB children to live in a more chaotic environment than TD children. Regarding temperament, we expected differences between the two groups mainly with regard to the emotionality dimension. We then considered all these factors together to see which combination of them best accounted for the difference between the two groups of children.

## Method

### Participants

Children from the EB group were recruited by informing pediatricians, schools and the media of our research. Parents who felt that their child presented EB problems and who were interested in participating in the study registered online. They were asked to complete the Child Behavior Checklist (CBCL; Achenbach & Rescorla, 2000). From this questionnaire, we considered the EB scale and used the cut-off score of 21, as this is the point at which a child is considered to present a borderline level of EB (Achenback & Rescorla, 2000). Only children with a score of 21 or above were selected for consideration in the EB group. Children from the TD group were recruited in preschool classes after checking with the CBCL that their EB score was below 21. Forty-nine children with externalized behavior problems (EB children) and forty-nine typically developing children (TD children) matched to the EB children in terms of chronological age and gender were thus recruited. Parents received an information letter and a consent form for the participation of their child in the study. Children were between 40 and 70 months old (*M* age = 59.57 months, SD = 6.77). There were 24 girls and 25 boys in each group. Each child was tested in a quiet room for 90 minutes, at school for the TD group, in the university offices for the EB group.

### Instruments

#### Instruments for the inclusion criteria

##### Child Behavior Checklist (CBCL)

The Child Behavior Checklist 1^1/2^–5 is a list of statements about the child’s everyday behavior, rated on a Likert scale ranging from 0 to 2, with 0 meaning “not applicable,” 1 meaning “applies more or less or sometimes” and 2 meaning “always applicable”. The EB scale is the sum of the “Aggressive Behavior” subscale, which comprises 19 items, and the “Attention Problems” subscale, comprising 5 items. The CBCL scales have a Cronbach’s alpha between .63 and .86, and test-retest reliability is .85 (Achenbach & Rescorla, 2000). A score below 21 on the EB scale is considered “normal”.

##### Wechsler Preschool and Primary Scale of Intelligence (WPPSI-III)

We used two subscales of WPPSI-III (Wechsler, 2004) to evaluate IQ: “Information” from the verbal scale; and “Block design” from the performance scale. The mean of these two subtests is 10, with a standard deviation of 3. Children were included in the study if the mean of the standard score of these two subscales was between 5.5 and 14.5.

#### Demographic variables

Parental level of education was evaluated using a seven-point scale from low (incomplete elementary school) to high (university) education. Within the whole group, the mean for the mothers’ level of education was 5.09 (SD = 1.50), and 4.83 for the fathers’ (SD = 1.65), a score of 5 corresponding to short-term higher education. For monthly income (including any source of net income, for both parents), we used a nine-point scale from low (0–500 euros) to high income (more than 4000 euros). The mean was 6.61 with a standard deviation of 2.09, which corresponds to a middle class income of 2500–3000 euros a month.

#### Externalizing Behaviors Measures

##### Conners Rating Scale

We asked parents and teachers to fill in the Conners Parent and Teacher Rating Scale (CPRS, CTRS) for each child (Goyette, Conners, & Ulrich, 1978). These questionnaires, measuring parents’ and teachers’ perceptions of the child’s hyperactivity, inattention, impulsivity and conduct disorders, are composed of 48 items in the parent version and 28 items in the teacher version. Catale, Geurten, Lejeune, and Meulemans (2014) validated the factorial structure of the parent questionnaire and confirmed the good psychometric qualities. Indeed, the three main scales (conduct problems, learning difficulties and impulsivity/hyperactivity) present a Cronbach alpha between .76 and .80. In this questionnaire, adults must choose whether the statement represents a common behavior of the child (four-point Likert scale from “not at all” to “very much”). In this study, we used inattention, hyperactivity/impulsivity and conduct disorder factors, which are calculated as the sum of the respondents’ ratings of the relevant observed behaviors. T-scores (mean of 50, SD of 10) are then calculated and taken into account in our analysis.

##### Unfair Card Game (UCG)

The Unfair Card Game (Roskam et al., 2016) is inspired by an adult paradigm focusing on perspective-taking (Bukowski & Samson, 2016) and is based on a cooperative computer game where the child is invited to play with a virtual child named Thomas. It has been designed to induce spontaneous positive affects in the first part and frustration in the second part. The game is presented to the child as one where he/she can win candy. The child sits next to the examiner at a table facing the computer. When the game starts, instructions are given to the child by a virtual examiner (a previously video-recorded adult). Two cards are shown on the screen; on one of them there is a picture of a piece of candy. Then the cards turn over and start to move. When the cards stop moving, the child must indicate which is the card with the candy. The child is invited to play five rounds. For each correct answer, he/she gives a piece of candy to Thomas, his virtual partner. After the first five rounds, it is time for Thomas to play. It is explained that a candy will be given to the child for each of Thomas’s correct responses. However, the game is rigged such that the child wins his/her five rounds and therefore Thomas wins five pieces of candy (this is called the winning phase), but Thomas wins only one round, so the child receives only one piece of candy (losing phase). At the end of the game, Thomas tells the child that he played badly and that he will therefore share his candies with the child. In this way, the child’s level of frustration returns to normal. This game lasts for 10 minutes. The advantage of this observational paradigm is that we can control the reaction of the adversary, as each child is faced with the same virtual partner (Thomas). The examiner’s speech is also strictly standardized (comments made at the end of each round, for the two phases). The UCG is video-recorded and coded following standardized guidelines. Four dimensions are coded: positive affect (smile, laughter, etc.) negative affect (tears, insults, etc.), agitation (movements) and inattention (distraction). For each of these dimensions, frequency and intensity are taken into account when coding, using a 5-point Likert scale ranging from 1 (neither frequent nor intense) to 5 (very frequent and intense). Coding was done by trained coders. The intercoders’ reliability, calculated with the weighted Kappa coefficient, reaches .766.

#### Executive Functions Measures

##### Inhibition/Flexibility

###### Cat-Dog-Fish

The cat-dog-fish task (Noël, unpublished) is a task inspired by the Day/Night test (Gerstadt, Hong, & Diamond, 1994) which assesses inhibitory control. There are two conditions: in the *control condition*, a card of 24 drawings (cats, dogs and fish) is presented to the child. He/she must name the pictures on the card as quickly as possible and without error. In the *inhibition condition*, we tell the child that, on Mars, “cats” are called “dogs”, “dogs” are called “cats” and fishes are called fishes (in French, the word for “cat” (chat) is very close phonologically to the word “dog” (chien)). The child is invited to follow the new rule and give the “Martian” animal names for the animals on the second card as fast as possible and without error. The reliability of this test measured by Cronbach’s alpha is excellent for the inhibition condition (.92) (Volckaert & Noël, 2015). Our measure is an efficiency score for the inhibition condition, i.e., precision (number of correct responses) divided by time (in seconds), so that a high efficiency score corresponds to a good performance.

###### Fish

Fish is a Simon task that was developed with E-Prime Software (for more information see Schneider, Eschman & Zuccolotto (2002) E-Prime User’s Guide. Pittsburgh: Psychology Software Tools, Inc.). A fish appears on the computer screen and the child is asked to push on the side toward which the fish looks, regardless of on which side it appears. The response keys correspond to the letters S and L on an AZERTY keyboard. There were two types of stimuli: congruent or incongruent. In the case of congruent stimuli, the fish appears on the right and looks to the right OR the fish appears on the left and looks to the left. In the case of incongruent stimuli, the fish appears on the right and looks to the left OR the fish appears on the left and looks to the right. A practice block of 8 items preceded the test itself, which includes 40 items. Items are pseudo-randomly intermixed, with the constraint that the same response (S or L) cannot occur twice, nor the same condition (congruent or incongruent). Fish remain on the screen until the child responds. As we developed this task, its reliability was measured by calculating Cronbach’s alpha on data collected and the result is excellent (.91). An efficiency score for the incongruent condition was calculated by dividing precision (correct responses) by time (in seconds).

###### Head-Toes-Knees-Shoulders (HTKS)

This task was developed by Ponitz, McClelland, Matthews, and Morrison (2009). In its original form, it is composed of three parts, but we have added a fourth. In the first part, the child is asked to touch his/her head when the examiner says “touch your feet”, and to touch his/her feet when the examiner says “touch your head”. In the second part, shoulders and knees are added. The child must now touch his/her knees when the examiner says “touch your shoulders” and vice versa, in addition to the two instructions in Part 1. In the third part, the rules are changed: the child must now touch his/her knees when the examiner says “touch your head” and touch his/her shoulders when he says “touch your feet” (and vice versa). This third part is administered only if the child has correctly answered at least 5 of the 10 items in Part 2. We created a fourth part, which is always performed, in order to test flexibility. In this part, there are two hoops on the floor, a red one and a blue one. When the examiner is in the blue hoop, the child has to do what the examiner says (i.e., to touch his/her head when the examiner says “touch your head” and to touch his/her feet when told to do so). However, when the examiner is located in the red hoop, the child must do the opposite and touch his/her feet when told to touch his/her head (and vice versa). At the beginning of each part, there are 8 practice items to ensure that the child understands the rule. The number of correct responses for each part is calculated. For the inhibition parts, we used the number of correct responses for the three first parts. For flexibility, we used the number of correct responses for Part 4.

##### Attention

###### Cats

The *cats* task is a cancellation task from the NEPSY battery (Korkman, Kirk, & Kemp, 1998) measuring selective visual attention. The internal consistency is good (.71) and the test-retest stability correlation is .62. In this task, the child had to cancel as many cats as possible without paying attention to distractors. Maximum duration is 180 seconds. The child is asked to be as fast as possible. Our measure is an efficiency score, taking into account precision (number of correct responses minus errors) and time (in seconds).

###### Auditory Attention

In this task from the NEPSY battery (Korkman et al., 1998), the child listens to an audio recording and has to put a red square in a box when and only when he/she hears the word “red”. The internal consistency is good (.81) and the test-retest stability correlation is .81. The precision score used in this study is calculated by subtracting errors from correct responses.

###### Reaction Time Variability

For each child we calculated the Reaction Time Variability (RTV) from the Fish task items, because it was the only task for which we had reaction time (RT) for each of the 40 items. The RTV was measured using the coefficient of variation, i.e., the ratio between the standard deviation of the RT (for the successful items only) and the RT median (again for the successful items only) (Kofler et al., 2013).

##### Working Memory

###### Word span

The Word Span task (Noël, 2009) was used to assess verbal short-term memory (phonological loop). In this task, the examiner presents a series of words to the child (one per second), who is asked to repeat them in the same order. The first level of difficulty includes two words; one more word is added for each new level. Each level of difficulty has three trials, and if the child fails in at least two out of the three trials, the task is stopped. We used the corrected span as the dependent measure: this is the longest sequence for which two series were repeated correctly, plus .5 if one longer series was also correctly processed.

###### Block tapping test

The Block Tapping Test (Noël, 2009), initially developed by Corsi (1973), is a measure of short-term memory of visuospatial information (visuospatial sketchpad). The examiner and the child sit face to face with a board between them, onto which are glued nine identical cubes. The child has to imitate the path of the examiner, who touches sets of cubes of increasing number. There are, as for the word span task, three trials per level. Once again we used the corrected span as the dependent measure.

###### Categospan

This complex span task (Noël, 2009) was used to assess the central executive. The examiner orally presents one-syllable food or animal words which the child must then repeat by category, first naming the food items, then the animals. Trials with items drawn on cards are performed first to ensure that the child understands the instructions, and pictures of a forest and a plate are presented to the child to help recall animal and food names, respectively. There were three trials per level, with trial set length increasing in each level. We used the corrected span as the dependent measure.

##### Executive functioning questionnaire

###### Childhood Executive Functioning Inventory (CHEXI)

We asked parents and teachers to fill out the French version of the Childhood Executive Functioning Inventory (Catale, Lejeune, Merbah, & Meulemans, 2013) which evaluates the executive functioning of the child. It is composed of 24 items focusing on two factors, inhibition and working memory, and is scored on 1-to-5-point Likert-type scales. A mean score is calculated for each factor. For this French version, the authors found good internal consistency (Cronbach’s alpha was .85 for the inhibition subscale and .89 for the working memory subscale) and high test-retest reliability for the two subscales (.87 for the inhibition subscale and .75 for the working memory subscale).

##### Household chaos

###### The Confusion, Hubbub, and Order Scale (CHAOS)

We asked parents to fill out the CHAOS questionnaire (Matheny et al., 1995) to evaluate household chaos. This questionnaire contains 15 items to be answered by “true” or “false”. A total score is found using the sum of the 15 items’ answers. A higher total score represents a more chaotic household. The coefficient alpha for the 15 items is .79, and the test-retest stability correlation for the total score is .74 (Matheny et al., 1995).

##### Temperament

###### Colorado Childhood Temperament Inventory (CCTI)

The CCTI is a questionnaire created by merging the NYLS and the EASI scales (Rowe & Plomin, 1977). All scales present a Cronbach alpha between .73 and .88, and test-retest reliability is between .43 and .80. In this study, parents are asked to fill in twenty-five items of the CCTI about their child’s temperament and five scales are taken into account: Emotionality, Sociability, Activity, Soothability and Attention Span Persistence.

## Results

In this study, we first checked whether parents’ ratings of the child’s EB correlated with the teachers’ evaluations, as well as with the clinician’s observation of the child’s behaviour in the observational paradigm. Next, we compared the EB and the TD samples of children on the different dimensions that were measured: EF, RTV, temperament and chaos. Finally, all these variables were included in a discriminant analysis with the group as the dependent variable.

### Between group comparisons

Table 1 presents means and standard deviations for all variables. For all measures, *t*-test comparisons were conducted. The level of significance for all tests was set at 0.05. Effect sizes were calculated using Cohen *d* (see Table 1). We reported the number of children from each group for each variable in this table, as for some variables some subjects were not tested. Despite these differences in group size, the subgroups considered never differed in terms of age or gender. Children in the EB group did not differ from TD in terms of IQ, in terms of mother’s and father’s level of education, or in monthly income (see Table 1). We thus had perfectly comparable groups.

**Table 1:** Mean scores and standard deviations for each group and between-group comparisons.

		Pretest						

			TD group (N = 49)		EB group (N = 49)	Analysis *t*		Effect size *d* Cohen (r)

Variables		*N*	*M (SD)*	*N*	*M (SD)*			

***Inclusion criteria***	CBCL EB	49	8.95 (5.45)	49	28.21 (6.09)	–16.498	***	–3.3 (–.86)
	CBCL AttProb	49	1.72 (1.52)	49	5.16 (2.00)	–9.533	***	–1.94 (–.70)
	CBCL AggBehav	49	7.21 (4.46)	49	23.05 (4.93)	–16.684	***	–3.37 (–.86)
	Mean of IQ subtests	49	8.71 (2.14)	49	9.40 (2.36)	–1.504		
***Demographic datas***	CA (in months)	49	60.71 (5.00)	49	58.43 (8.06)	1.687		
	Mother education (max = 7)	48	4.92 (1.57)	48	5.27 (1.43)	–1.158		
	Father education (max = 7)	47	4.68 (1.64)	43	5.00 (1.66)	–.915		
	Family income (max = 9)	25	6.24 (1.69)	42	6.83 (2.28)	–1.127		
***Household Chaos***	CHAOS	26	2.42 (2.27)	44	6.25 (2.90)	–5.760	***	–1.47 (–.59)
***Temperament***	CCTI EMO	26	13.65 (3.70)	43	17.74 (3.29)	–4.777	***	–1.17 (–.50)
	CCTI ACTI	26	15.46 (3.67)	43	18.70 (3.00)	–3.986	***	–.97 (–.44)
	CCTI SOC	26	18.31 (3.54)	43	16.58 (4.58)	1.645		
	CCTI ATT	26	16.54 (2.87)	43	13.00 (3.87)	4.031	***	1.04 (.46)
	CCTI SOOTH	26	16.88 (3.20)	43	13.72 (2.88)	4.237	***	1.04 (.46)
***Behavioral measures***								

	CPRS conduct problems	26	41.6 (7.92)	44	62.68 (14.24)	–6.816	***	–1.83 (–.67)
	CPRS hyperactivity	26	43.88 (6.17)	44	68.05 (13.38)	–8.660	***	–2.32 (–.76)
	CPRS impulsivity	26	43.50 (6.17)	44	64.52 (10.01)	–9.662	***	–2.53 (–.78)
	CPRS learning problem	26	46.50 (9.57)	44	64.66 (17.76)	–4.807	***	–1.27 (–.54)
	CTRS conduct problems	27	46.44 (5.03)	35	55.00 (8.85)	–4.490	***	–1.19 (–.51)
	CTRS inattention	27	45.56 (4.76)	35	47.09 (5.71)	–1.123		
	CTRS hyperactivity	27	44.52 (5.18)	35	51.83 (8.06)	–4.100	***	–1.08 (–.47)
	UCG positive affects	49	1.51 (.48)	49	1.24 (.41)	2.895	**	.60 (.29)
	UCG negative affects	49	1.44 (.45)	49	1.63 (.62)	–1.733	^†^	–.35 (–.17)
	UCG agitation	49	1.70 (.64)	49	3.21 (1.05)	–8.619	***	–1.74 (–.66)
	UCG inattention	49	1.98 (.68)	49	2.18 (.80)	–1.291		
***Cognitive measures***								

*Attention*	Cats (ES)	48	.27 (.09)	49	.26 (.12)	.211		
	Auditory Attention	48	27.23 (10.45)	46	23.96 (14.41)	1.264		
*Working Memory*	Word Span	48	3.32 (.63)	49	3.13 (.62)	1.499		
	BTT	48	3.53 (.68)	49	3.16 (.81)	2.428	*	.49 (.24)
	Categospan	48	2.44 (.79)	48	2.32 (.82)	.697		
	CHEXI WM Parent	47	2.19 (.67)	47	2.56 (.66)	–2.727	**	–.56 (–.27)
	CHEXI WM Teacher	49	1.92 (.84)	38	2.30 (.92)	–2.040	*	–.43 (–.21)
*Flexibility*	HTKS 4	48	15.44 (3.95)	49	14.12 (3.63)	1.708	^†^	.35 (.17)
*Inhibition*	HTKS 1-2-3	48	31.69 (13.91)	49	26.27 (17.28)	1.700	^†^	.35 (.17)
	CDF inhibition condition (ES)	49	.52 (.17)	49	.42 (.19)	2.832	**	.55 (.27)
	Fish incongruent condition (ES)	27	.01 (.01)	44	.01 (.00)	2.872	**	0 (0)
	CHEXI Inhib Parent	47	2.73 (.68)	47	3.69 (.65)	–7.012	***	–1.44 (–.59)
	CHEXI Inhib Teacher	49	2.10 (.86)	38	3.07 (.90)	–5.106	***	–1.10 (–.48)
RTV	RTV Fish	27	.55 (.30)	44	.77 (.41)	–2.4	*	–.61 (–.29)

*Notes*: TD = Typically Developing; EB = Externalized Behavior; M = Mean; SD = Standard Deviation; CA = Chronological Age; IQ = Intellectual Quotient; CCTI = Colorado Childhood Temperament Inventory; EMO = Emotionality; ACTI = Activity; SOC = Sociability; ATT = Attention Span Persistence; SOOTH = Soothability; CBCL = Child Behavior CheckList; UCG = Unfair Card Game; CPRS = Conners Parent Rating Scale; CTRS = Conners Teacher Rating Scale; ES = Efficiency Score; BTT = Block Tapping Test; CHEXI WM = Working Memory scale of the CHEXI; HTKS = Head-Toes-Knee-Shoulder; CDF = Cat-Dog-Fish; CHEXI Inhib = Inhibition scale of the CHEXI; RTV = Reaction Time Variability. ^†^*p* < .1 *p ≤ .05, **p ≤ .01, ***p < .001.

### Behavioral measures

**Figure 1:**
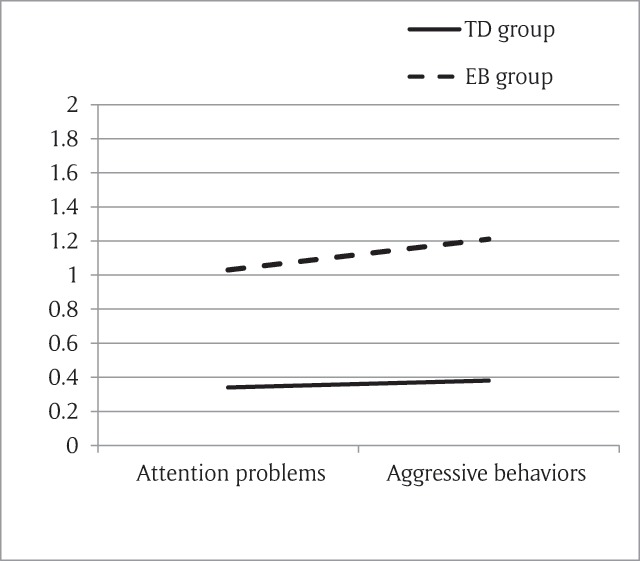
Difference between TD and EB groups on CBCL scales.

As the CBCL score was the criterion for selecting the two groups, differences between the two groups are trivial. However, we wondered if the difference between the two groups on the EB scale was more attributable to the “attention problem” scale or to the “aggressive behaviour” scale. Accordingly, we ran a repeated measures ANOVA with one within-subject factor, the scale (“attention problems” and “aggressive behaviors”), and one between-subjects factor, the Group (TD and EB group). As the two scales did not include the same number of items, we used a mean score for each of the scales (total score for the scale divided by the number of items of the scale). The results showed significant main effects of scale (*F*(1,96) = 10.940, *p* = .001, η_p_^2^ = .102) and group (*F*(1,96) = 206.467, *p* < .001; η_p_^2^ = .683) and a significant interaction between the two (*F*(1,96) = 5.244, *p* = .024, η_p_^2^ = .052). Even if the two groups differ for each scale (*t*(96) = –9.533, *p* < .001 for attention problems scale; *t*(94) = –16.683, *p* < .001 for aggressive behaviors scale), the difference is larger for the aggressive behaviors scale (see Figure 1). On the Conners scales, parents of the EB children rated their children as presenting more difficulties on all four scales (conduct problems, hyperactivity, impulsivity and learning problems) relative to parents of the TD group. Congruently, teachers rated EB children as presenting more conduct problems and more hyperactivity than TD children. However, teachers did not highlight inattention problems (see Table 1). In the observational paradigm, *t*-tests showed that children from the EB group were significantly more agitated than those of the TD group and presented significantly less positive affects, but the difference between the two groups was marginal for the negative affects and we did not find any difference for inattention (see Table 1).

**Figure 2:**
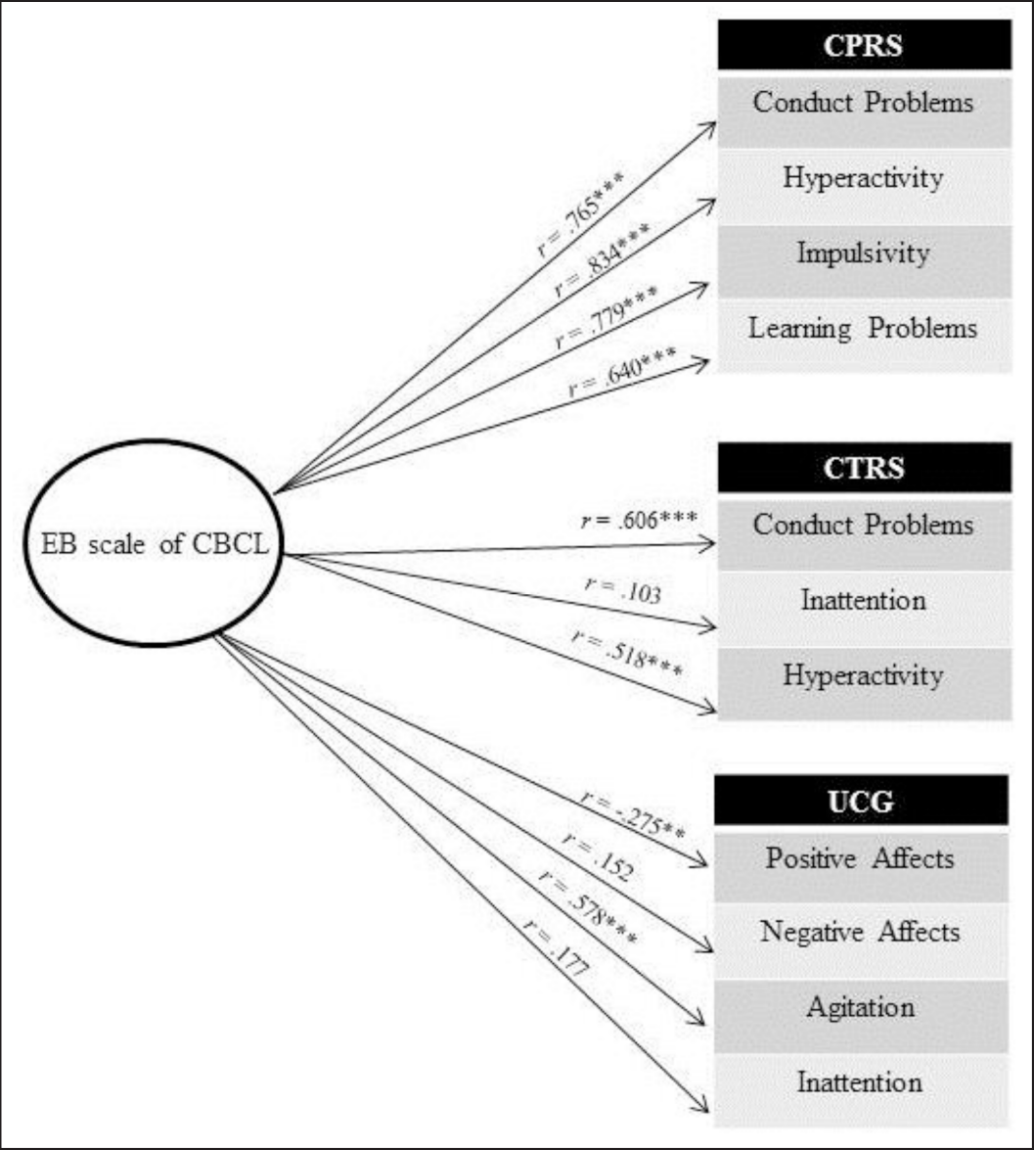
Intercorrelations among EB scale of CBCL and behavioral variables. *Notes*: CBCL = Child Behavior CheckList; UCG = Unfair Card Game; CPRS = Conners Parent Rating Scale; CTRS = Conners Teacher Rating Scale; UCG = Unfair Card game. *p ≤ .05, **p ≤ .01, ***p < .001.

Correlations were measured between the different ratings of the children’s behavior by the parents and by the teachers through questionnaires and by the clinician in the observational paradigm. As we see in Figure 2, the EB score of CBCL-Parents correlates very well with parents’ rating of all the Conners scales, i.e., conduct problems, hyperactivity, impulsivity and learning problems. More importantly, it also correlates with Conners scales teacher’s ratings of conduct problems and hyperactivity, which indicates that children’s EB were independent of the person rating the child’s behaviour and were not restricted to one specific environment. Finally, we also observed significant negative correlations between the EB score of CBCL-Parents and two scales of the UCG, i.e., positive affects and agitation, which seems to show that children’s EB are independent of the modality of the evaluation or of the evaluator.

### Temperament

As we see in Table 1, the EB group presents significantly lower attention capacities and greater difficulties in being soothed than the TD group. They also have a higher score on emotionality and activity than the TD group, which means that EB children have more difficulty managing their emotions and are more restless than TD children. However, we did not find any significant difference between the two groups on the sociability scale of the CCTI.

### Household Chaos

Despite the similarities in demographic variables, more household chaos was characteristic of the EB group (see Table 1). Indeed, although there are no normative data for this questionnaire (the higher the score, the higher the household chaos), we observed that nearly all the TD children (96%) had a chaos score below the midpoint 7/15, while 40% of the EB children had a total score above that midpoint.

### Cognitive measures

The TD group outperformed the EB group on all the *inhibition tasks*, except for the HTKS where the difference was marginal. Concerning the *working memory tasks*, the two groups differed significantly only on the visuospatial sketchpad, with an advantage for the TD group (no difference for the phonological loop or the central executive). For *flexibility* (fourth part of HTKS), the difference between the two groups did not reach significance. Concerning *attention*, we did not find any difference for either visual or auditory tasks. Finally, on the two scales of the CHEXI, both parents and teachers judged the EB children to have lower working memory and lower inhibition capacities relative to TD children.

In the same vein as Nigg, Willcutt, Doyle, and Sonuga-Barke (2005), we wanted to further explore the number of failed tasks by each child in each group to see to what extent these cognitive tasks might help in the diagnosis of EB. To this end, we computed a z-score for each of the cognitive tasks and considered that a child failed a task when the z-score was below 1.5. Only 2% and 4% of the TD and EB children respectively failed at least one of the attention tasks. For working memory, this percentage reached 18.4% for both the TD and EB children. The flexibility task was failed by 12.5% of the TD children and 10.2% of the EB children. The profile of the two groups differed only for the inhibition tasks: 18.4% of the TD and 47% of the EB children failed at least one of the inhibition tasks (X(1) = 9.095, *p* < .003).

Our last cognitive measure was the RTV, which showed that the EB group presented a higher variability in reaction time than the TD group (*t*(69) = –2.400, *p* = .019).

### Discriminant analysis

A discriminant analysis was then run to see how these different dimensions might help to distinguish between the EB and the TD groups. In order to work with more global measures, factorial analyses in principal components forcing to one factor were computed to obtain a single score for, respectively, the working memory, the inhibition and the attention dimensions, as well for temperament (CCTI). As can be seen in Table 2, the attention factor was calculated on the cats and auditory tasks. The saturation of each task was .856 and the factor accounted for 73.3% of the variance. The loading for the three working memory tasks (categospan, word span, block tapping test) ranged between .674 to .838 and the factor accounted for 57.4% of the variance. The inhibition factor was calculated on the fish, cat-dog-fish and HTKS tasks. The saturation of tasks on this factor ranged from .770 to .783 and the factor accounted for 60% of the variance. The last factor was calculated on scales of CCTI (emotionality, attention, soothability, activity). The saturation of tasks on this factor ranged from .601 to .839 and accounted for 50.8% of the variance.

**Table 2:** Tasks loadings for the two factors resulting from the factorial analysis.

Tasks	Loadings on the factor

***Factorial analysis for Attention***	

Cats	.856
Auditory attention	.856
**% of explained variance**	**73.3%**
***Factorial analysis for Working Memory***	

Categospan	.838
Words span	.751
Block Tapping Test	.674
**% of explained variance**	**57.4%**
***Factorial analysis for Inhibition***	

Fish	.783
Cat-Dog-Fish	.771
HTKS	.770
**% of explained variance**	**60%**
***Factorial analysis for Colorado***	

Emotionality	.839
Attention	–.739
Activity	.649
Soothability	–.601
**% of explained variance**	**50.8%**

These four factors (attention, working memory, inhibition and temperament) were then entered, together with the CHAOS (total score), flexibility (correct responses for the fourth part) and RTV measures, as independent variables in a discriminant analysis with the group (EB or TD) as the dependent variable. The obtained discriminant function was significant (Wilks’s lambda of .39, χ^2^_(7)_ = 56.80, *p* < .001, R^2^ = .61). However, Table 3 shows that attention, working memory and flexibility did not contribute to discriminate the two groups. We thus ran another discriminant analysis removing those three variables. This new discriminant function involving CCTI, Chaos, RTV and inhibition factor was significant (Wilks’s lambda = .44; χ^2^_(7)_ = 52.16, *p* < .001, R^2^ = .56) and made it possible to correctly classify 84% of the TD children and 88% of the EB children. It is however important to note that the primary explanatory factor is temperament (CCTI), which accounts for 40% of the variance; then chaos, which adds another 8% of explained variance; then RTV, which adds another 6%; and, finally, inhibition factor, which contributes to another 2% of supplementary explained variance.

**Table 3:** Tests of Equality of Group Means.

	Wilk’ Lambda	F	df1	df2	Sig

Colorado	.590	43.799	1	63	<.001
CHAOS	.620	38.652	1	63	<.001
RTV	.899	7.080	1	63	.010
Inhibition	.953	3.107	1	63	.083
Flexibility	.981	1.188	1	63	.280
Working memory	.993	.462	1	63	.499
Attention	1.000	.014	1	63	.905

## Discussion

Research into EB has grown in the literature over the last few years, highlighting several risk factors for developing EB at a very young age. But studies which investigate many of the aspects of life which are affected by EB in this population of EB preschoolers are rare. In this study, we wanted to observe the profile of EB preschoolers, taking into account the behavioral, cognitive and environmental spheres. We first wanted to observe whether children rated by parents as presenting EB would also be rated in this way by teachers, and whether the differences in EB between the two populations would also be apparent in an observation paradigm. We then compared our two groups on cognitive tests, temperament and household chaos. Finally, all these dimensions were integrated into a discriminant analysis in order to examine their relative power in predicting group belonging (EB or TD). Before further consideration, we need to make the following point. It is usual in the literature to point out that EB children often come from low SES families (Bradley & Corwyn, 2002; Hill, 2002). In this study, however, the two samples compared did not differ in terms of family income or parental educational level.

As we created our groups on the basis of the CBCL parental rating of EB and as we know the importance of multi-informant evaluation (Roskam et al., 2011), we first examined whether EB children are different from TD children not only in parental evaluations of their behavior but also in teachers’ ratings. EB children are generally difficult both at home and at school, as in any other environment and this is exactly what we found in this study. EB children were also rated by parents as presenting more conduct problems (and learning disabilities), and as being more hyperactive and impulsive. Moreover, parents’ ratings of EB positively correlated with Conners teachers’ evaluations, and we observed that EB children were also rated by teachers as presenting more conduct problems and as more hyperactive.

Secondly, as Roskam et al. (2011) also insist on the importance of multi-method evaluation, we expected that our two groups would not only differ on questionnaires but also in an observational paradigm, i.e., the UCG. Our analysis showed that EB children were indeed more agitated than TD children and presented fewer positive affects. We also observed a tendency in EB children to show more negative attitudes to the situation of frustration than TD children.

Third, as we know there is a link between EB and EF in preschoolers (Pauli-Pott & Becker, 2011; Schoemaker et al., 2013), we expected to observe differences on cognitive variables between the two groups, especially on inhibition capacities. Indeed, on the CHEXI questionnaire, EB children were rated by both parents and teachers as presenting lower inhibition capacities than TD children. Moreover, in objective tasks, they presented weaker performance for cognitive and motor inhibition. Concerning flexibility and working memory, the literature shows weaker correlations with EB (Pauli-Pott & Becker, 2011; Schoemaker et al., 2013). Consistent with this, our results showed that there was a tendency for the TD group to perform better on the flexibility task, while for working memory, we observed a weaker performance for the EB relative to the TD groups only on the block tapping test, which evaluates the visuospatial sketchpad of short-term memory, but not on the two other tasks. Even if some research in school age children has shown that a central executive deficit may be characteristic of ADHD (Karatekin, 2004), our results are in line with those authors who suggest that visuo-spatial working memory may be a sensitive measure for ADHD (R. Barnett et al., 2001; Westerberg, Hirvikoski, Forssberg, & Klingberg, 2004). Regarding attention, the two populations did not differ on either of the two attention tasks, whatever the modality (visual or auditory). This is surprising given the significant correlation found in the literature between attention capacities and EB (Pauli-Pott & Becker, 2011). One explanation could be that at this very young age, the predominant symptoms of EB reported by parents or even teachers are agitation and hyperactivity/impulsivity, as the children have difficulties sitting still, and that attentional symptoms are more symptomatic of the school-age period, as this is the phase during which children must stay focused to listen to the teacher, to instructions, etc. However, in this study, we observed that parents rated EB children as presenting more attention problems on the CBCL questionnaire. Another explanation could be related to the sensitivity of the tests used. Indeed, as both attention tasks were quite short (only three minutes each), perhaps this duration is insufficient to highlight attentional lapses. It is, however, important to note that we observed a greater RTV in the EB group than the TD group. One might perhaps argue that RTV is therefore a more sensitive measure of attention lapses than the two attentional tasks. Future studies should use more sensitive attention tasks as the Attention Network Test (ANT) (Rueda et al., 2004), which can also measure RTV, and take account of all attentional components.

Our fourth hypothesis concerned household chaos. The literature has shown correlations between family environment and the presence of EB (Corapci & Wachs, 2002; Farbiash et al., 2014). Our results confirmed these findings, since we found that families of EB children present more household chaos than families of TD children.

Finally, we investigated the temperament of each population. Research shows that it is mostly emotionality which is correlated with the emergence of EB (Schmitz et al., 1999). In this study, we found that emotionality significantly distinguished the two groups, but that the scales of soothability, activity and attention also showed significant differences. The only dimension which did not distinguish the two groups was sociability. The failure to find any difference at that level is surprising, as we know that EB children often have weaker social competences (Nader-Grosbois, Houssa, & Mazzone, 2013). One explanation could be that, as CCTI is a questionnaire relating to temperament, the sociability scale items from CCTI (e.g., “my child is friendly with strangers”, “my child has a tendency to be shy”) refer more to extraversion than social competences *per se*. In this case, our results would be in line with the research of Meunier et al. (2012), who showed that extraversion was not related to child EB.

In this study, we thus show that EB children have certain specificities, not only in the behavioral sphere, but also in the cognitive and environmental areas. Our discriminant analysis also highlighted that temperament, household chaos, RTV and inhibition capacities were the best predictors of EB. Given these results, we believe that it could be interesting to consider those aspects in future clinical studies. However, none of these factors by itself could be taken as a diagnostic criterion of EB. This was illustrated with inhibition measures. Research, including this study, has revealed the link between inhibition and EB (Pauli-Pott & Becker, 2011; Roskam et al., 2013; Schoemaker et al., 2013) and our discriminant analysis showed that inhibition significantly contributed to explaining some part of the inter-group variance. However, only 47% of the EB children failed in one or more inhibition task, which means that 53% of these EB children did not fail any task. This result is not surprising. Indeed, EB problems are highly heterogeneous and different causes can lead to a EB profile. Inhibition is therefore just one of the many factors that should be considered in analyzing the profile of an EB child. Establishing this large profile would make it possible to identify the specific risk factors that the child is presenting and accordingly, to target more precisely the type of intervention needed. For instance, EB children who present inhibition impairments would probably benefit from neuropsychological interventions (see e.g., Volckaert & Noël, 2015), but other children presenting problems such as poor language or parenting, would need other types of interventions. This observation could also have an impact on the clinical practice of some therapists. Indeed, until now, neuropsychologists have mainly focused their evaluation on cognitive functions, whereas clinical psychologists focus more on the affective, family and environmental spheres. But with the profile that we have highlighted in this study, showing the presence of several risk factors, it would be more beneficial for the patient if therapists privileged a multidimensional approach. Such an approach could help the therapist detect the risk factors presented by each specific child and orient the intervention accordingly. For instance, parental guidance might be useful for a child living in a very chaotic environment while a neuropsychological intervention might be more appropriate for another child presenting very weak EF.

Although this research outlined a comprehensive profile of EB preschoolers which could be useful for clinical practice and intervention, several limitations need to be noted. First, our sample was small, although the two samples were very neatly matched in terms of chronological age and gender, and did not differ in terms of family income, level of education of the parents or the child’s IQ. Moreover, although our baseline was very complete, allowing us to investigate a large range of variables, it would have been interesting to add a measure of “hot” EF to our baseline. Zelazo and Müller (2002) proposed a model that distinguishes “hot” EF and “cold” EF: “hot” EF refers to the affective aspects of EF, related to decision-making, which leads to an emotional consequence such as gain or loss, and mostly involves the orbitofrontal area of the prefrontal cortex; “cold” EF refers to cognitive EF, which does not involve emotional arousal, is induced by abstract or decontextualized problems and mostly involves the dorsolateral area of the prefrontal cortex. Although these authors defend the idea of a deficit of cold EF in ADHD, others argue for an impairment of hot EF (Garon, Moore, & Waschbusch, 2006), or the possibility of two pathways, one related to cold or one related to hot executive function (the Dual Pathway Model of ADHD from Sonuga-Barke (2002)), so it would have been interesting to study the hot EF capacities in EB preschoolers. Concerning chaos, it would have been useful to have a measure that allowed us to highlight which part of the family environment might be responsible for the chaos. Measures like HOME (Bradley & Caldwell, 1977) allow this kind of analysis by using several scales of family chaos (e.g., learning opportunities, physical environment, emotional climate) and could be used in future studies.

In summary, we found that EB children differ from TD children, not only in the behavioral sphere, but also in cognitive and environmental areas, and that these characteristics can already be observed at preschool age. However, despite the differences between the two groups, it seems that EF performance has a weak power for EB diagnosis and that variables such as temperament or even household chaos best distinguish the two populations.

## Footnotes

The authors created an overall EF factor by computing the weighted mean effect size across all EF tasks in each studies of the meta-analysis.
